# Passive anti-leakage of blue light for phosphor-converted white LEDs with crystal nanocellulose materials

**DOI:** 10.1038/s41598-023-39929-2

**Published:** 2023-08-10

**Authors:** Quang-Khoi Nguyen, Benoit Glorieux, Gilles Sebe, Tsung-Hsun Yang, Yeh-Wei Yu, Ching-Cherng Sun

**Affiliations:** 1 Department of Optics and Photonics, National Central University, Chung-Li, 32001 Taiwan; 2 Falculty of Physics and Engineering Physics, VNUHCM-University of Science, Ho Chi Minh City, 700000 Vietnam; 3 Vietnam National University Ho Chi Minh City, Ho Chi Minh City, 700000 Vietnam; 4 Univ. Bordeaux, CNRS, Bordeaux INP, ICMCB, UMR 5026, F-33600 Pessac, France; 5 Univ. Bordeaux, CNRS, Bordeaux INP, LCPO, UMR 5629, F-33600 Pessac, France; 6 Department of Electrophysics, National Yang Ming Chiao Tung University, Hsinchu, 30010 Taiwan

**Keywords:** Lasers, LEDs and light sources, Optical materials and structures

## Abstract

A phenomenon known as "blue-light leakage" caused by overheating pcW-LEDs has recently been identified, and it poses a risk to users. This study focuses on investigating and optimizing a solution to address this issue. To tackle the problem of overheating and blue light leakage, we explored the application of a specific thermochromic material called crystal nano cellulose (CNC). We introduced CNC inside the epoxy lens of white LEDs. Importantly, under standard conditions, CNC has a negligible impact on the optical properties of the output white light. However, when overheating conditions arise, leading to blue light leakage, the temperature increase triggers a darkening effect in CNC. This thermochromic behavior of CNC allows it to strongly absorb the blue light, resulting in a significant suppression of the output luminous flux. As a result, the lamp dims, which not only prevents the user's eyes from being exposed to harmful bluish light but also serves as an indicator of aging in the pcW-LED. By implementing CNC as a responsive material in the design of white LEDs, this study offers a practical and effective solution to mitigate the negative effects of blue-light leakage caused by overheating. This improvement enhances the safety and comfort of users while also providing an early warning system for the aging of pcW-LEDs.

## Introduction

Solid-state lighting (SSL) using the white light source of phosphor-converted white light-emitting diodes (pcW-LEDs) has shown feature properties such as high energy efficiency, fast response, proper color rendering, long lifetime, and low cost^1–6^. The white light can be generated based on di-chromatic, tri-chromatic, and tetra-chromatic^2^. The most simple, efficient, and widely utilized method is using a blue LED die to excite the down conversion yellow phosphor of YAG:Ce, the output mixing of blue and yellow light will cause the “white” perception for human eyes^2^. Among the research related to the pcW-LEDs, problems of low spatial color uniformity distribution, low color rendering index (CRI), blue light hazard, temperature dependency of efficiency, and color performance has been attracted many scientists to study and reported through many articles^7–16^. Recently, the phenomena of blue leakage that occurs during the operation process of pcW-LEDs have been reported by Sun et al. in a report in 2022^17^. The blue leakage phenomenon is defined by a significant decrease in the yellow emission of the phosphor and a not-so-important decrease in the blue light coming from the die. So the correlated color temperature (CCT) value related to the Blue/Yellow ratio increases drastically. The reasons for bluish light output are related to the overheating effect that origins from the limitation of internal quantum efficiency and Stoke loss of the phosphor^18–20^. There are many reports about the effect of blue light on the retina tissue of human eyes^21–24^. Human eyes face risks regarding visualization, healthy lighting, and photobiology safety. Some effort has been made to reduce the negative effect of blue light on biological safety, visualization, and sleep quality for humans^25–28^. Several studies proposed solutions for the thermal management of pcW-LEDs. Yang et al. reported that based on self-compensation between the excitation efficiency and conversion efficiency of the phosphors, the stabilizing of the CCT in pcW-LEDs can be obtained^29^. In a related study, Yang et al. proposed a practical approach for measuring phosphor temperatures in operating pcW-LEDs which can help to have the information on thermal conditions for controlling the thermal effect in pcW-LEDs^30^. Sun et al. designed circuit protection for enhancing the photobiological safety of human eyes from the pcW-LEDs with happens overheating conditions^17^.

In recent years, the development of nanoscience and nanotechnology has opened many opportunities to find exciting solutions for solving emerging issues in solid-state lighting, like blue leakage. Cellulose is one of them. The cellulose family can be synthesized in different morphology and size, such as cellulose nanocrystals (CNC), cellulose nanofibers (CNFs), and bacterial cellulose (BC)^31–33^. Several studies reported on the application of CNC to solve some problems of pcW-LEDs applications. Xu et al. reported that the covering a nanocrystal-filled polymer layer outside of an encapsulant lens, the angular color uniformity of phosphor-converted white LEDs could be reduced by 71.4% while maintaining over 85% of light energy transmission, the reported value of angular CCT deviation (ACCTD) was 587 K at 4% CNCs^34^. Chowdhury et al. reported that filling CNC into the phosphor layer reduces angle-dependent CCT deviation. When simulated with pcW-LEDs at CCT of 4220 K, with a setting of filling CNC 3% and 6% of the weight, the obtained ACCTD was 173.45 K and 59 K, respectively^35^. To our knowledge, such a study using the CNC to prevent the blue light leakage issue still needs to be reported.

In this paper, we propose a solution to absorb the blue light when the blue light leakage happens. We first investigated CNC's thermal and optical properties to ensure it is suitable for applying blue light leakage in pcW-LEDs. Finally, we introduce CNC into the epoxy lens covering the die and the phosphor of pcW-LEDs. The effect on the optical properties is analyzed, specifically when the blue light leakage phenomenon appears.

## Working principle

**Figure 1:**
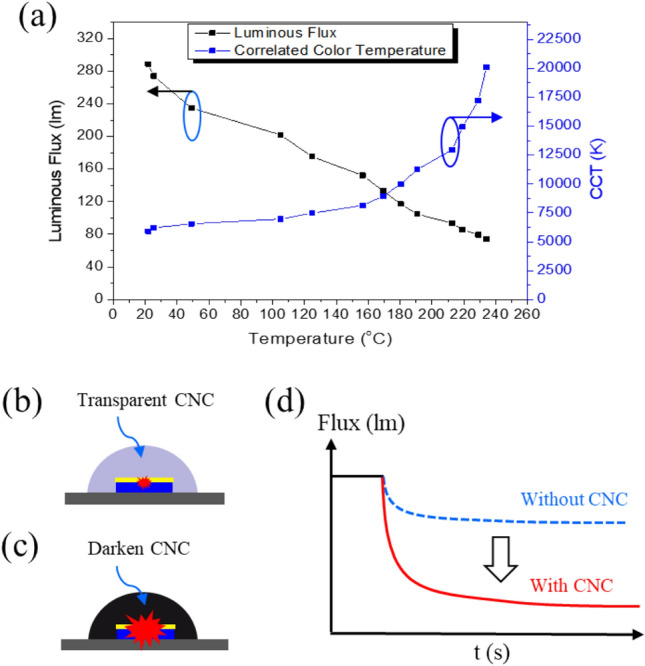
(**a**) Characteristic of blue leakage phenomena. (**b**) Illustration of color characteristics of samples at normal conditions, and (**c**) overheat conditions. (**d**) Illustration of luminous flux suppression caused by the darkening of CNC.

The blue light leakage issue is related to a variation of the blue and yellow light ratio (B/Y ratio) due to different effects of the thermal increase on blue light (coming from the die) and yellow light (coming from the phosphor). To analyze this phenomenon, an experiment was conducted where the overheat condition for pcW-LED was generated, and the optical properties were measured. In the experiment setup, the thermal couple (type T) is set at the backside of pcW-LEDs. Overheating is generated by less heat dissipation media when using the glass plate in the setup^17^. An integrating sphere is used to measure the behavior of optical properties. The result of the effect of overheating in causing the blue light leakage problem is shown in Fig. 1a. With the increase in temperature, the luminous flux is decreased. However, the value of CCT is increased correspondently, especially as the temperature is higher than 180 °C. The value of CCT depends on the B/Y ratio, so the increase in CCT value means an increase of the B/Y ratio, i.e., a blue leakage.

Our objective is to prevent blue leakages by introducing the thermochromic material of CNC inside the epoxy lens of pcW-LEDs. The working principle for preventing blue light leakage is based on CNC's optical and thermal behavior at normal and overheating conditions. At standard conditions, the temperature inside the pcW-LEDs is low (e.g., less than 150 °C). Since the temperature value is not high enough to cause the color change for CNC, the lens is still transparent, as shown in Fig. 1b, and there is no effect on the output white light. When blue light leakage appears, the temperature in the epoxy lens is higher than the stable temperature of CNC (e.g., higher than 200 °C), and thus CNC becomes dark, as shown in Fig. 1c. The darkened CNC will absorb the output light strongly, especially in the blue range. As a result, the output flux is significantly dimmed, as shown in Fig. 1d.

## Investigation of the properties of CNC

**Figure 2:**
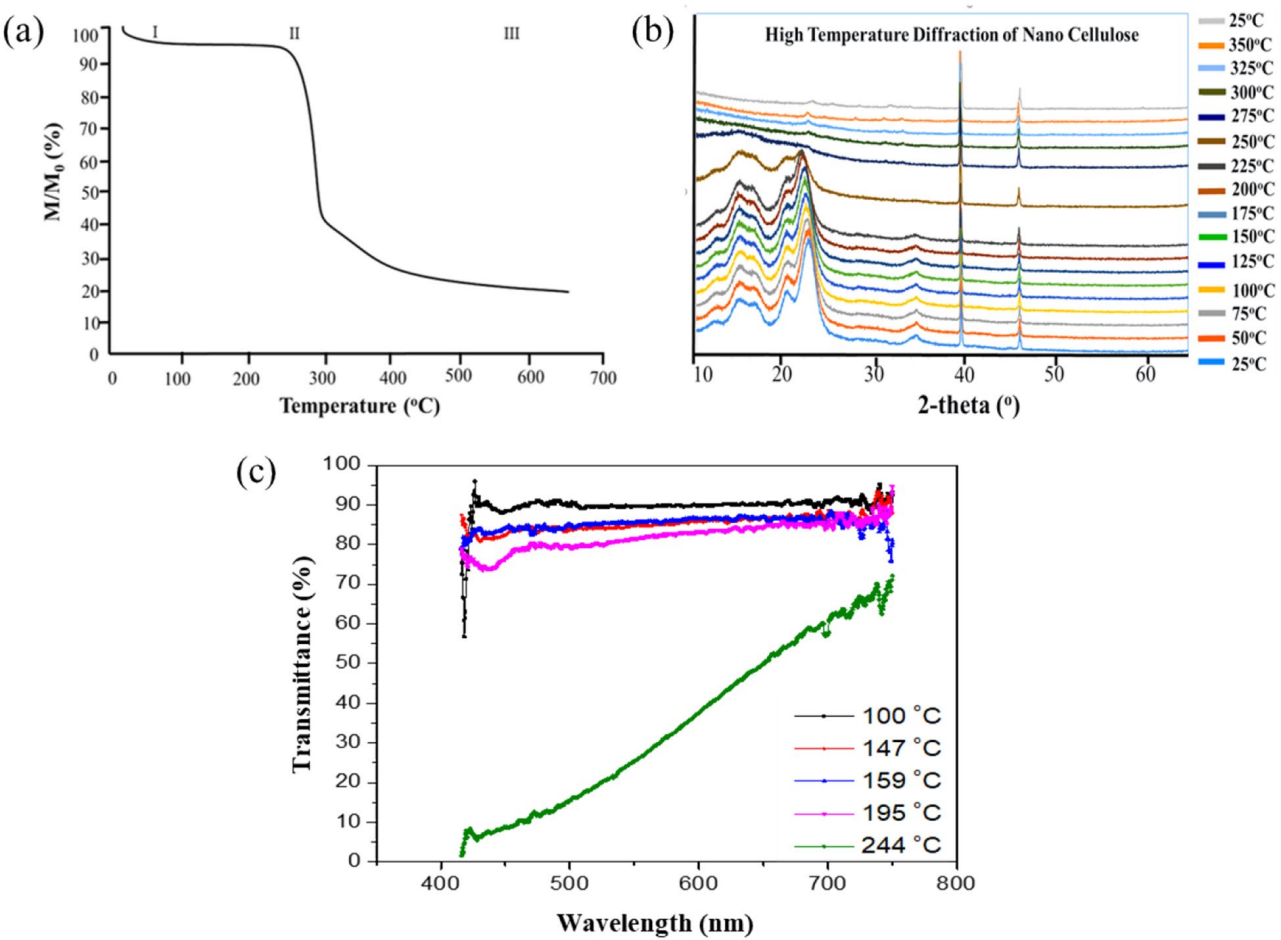
(**a**) TGA thermogram of the CNC-Chl particles between 20 and 650 °C. (**b**) X-ray diffraction spectra of the CNC-Chl particles at different temperatures. (**c**) Transmittance of the silicone plates filled with CNC-Chl at different temperatures.

CNCs are nanosized rod-like particles that are increasingly considered suitable for building blocks for elaborating materials with applications in areas as diverse as biotechnology, electronics, optics, and packaging^32,33,36^. In this work, CNCs provided by the University of Maine (Forest Products Laboratory) were used for their thermal properties as the optical fuse for pcW-LEDs devices ^37^. They consist of rod-shaped crystalline particles of 110 ± 48 nm in length and 4.8 ± 1.1 nm in thickness, as determined by atomic force microscopy in a previous study^38^. To favor their dispersion in the hydrophobic silicone matrix of the device, the CNCs surface was hydrophobized through the grafting of chloroacetate moieties (Chl), via an acylation reaction with vinyl chloroacetate (Sigma Aldrich). This reaction was carried out in anhydrous dimethylsulfoxyde (DMSO) with K_2_CO_3_ as a catalyst, according to an experimental protocol already reported in the literature^38,39^. After confirmation of the surface grafting by FTIR spectroscopy, the thermal properties of the CNC-Chl particles obtained were evaluated by ThermoGravimetric Analysis (TGA), high-temperature X-ray diffraction, and high-temperature transmittance. Results are shown in Fig. 2.

Thermogravimetric analysis was performed with a TGA Q50 (TA Instrument) equipment, at a heating rate of 10 °C/min and under an N2 environment. The thermogram of Fig. 2a is consistent with the three-step degradation process generally observed when CNCs are submitted to a temperature gradient^40^: first, the CNC loses the water adsorbed at their surface between 20 and 125 °C (I); then the CNC is decomposed into tarry volatiles (levoglucosan and anhydrosugars) and chars between 200 and 400 °C (II), by simultaneous depolymerization and dehydration reactions (decomposition starts at 206 °C, with a maximum rate of degradation at 274 °C); Finally, the aliphatic residue is converted into polycyclic aromatics between 400 and 600 °C (III).

High-temperature X-ray diffraction was performed on a PANalytical X'pert MDP diffractometer equipped with a backward monochromator, an Anton-Paar HKT16 chamber, a scintillation detector, and a Cu-Kα (l = 0.15418 nm) radiation generator. Measurements were performed from room temperature to 300 °C, with 25 °C increments, and at a heating rate of 3 °C/min. The last diffractogram at 25 °C was recorded after cooling down at room temperature. Data acquisition was performed in the 2θ range 10°–80°, with a step size of 0.017°. The patterns were analyzed using the Eva Bruker^®^ software and compared to the PDF database using the FindIt ICSD software. In the diffractograms, the two peaks at 39.5 and 46° belong to the aluminum sample holder; their slightly low theta shift is related to its thermal dilation. The broad pattern below 25° is related to the CNC-Chl sample and is typical of the cellulose I structure found in native cellulose^41^. These diffraction peaks are shifted towards lower angles below 200 °C without modification of the cellulose pattern, suggesting a dilatation of the CNC-Chl particles with no impact on the structure. A more careful analysis reveals that the shifts of the different peaks differ as a function of the temperature, indicating that the expansion is oriented in a specific direction. At 225 °C, the pattern of cellulose starts to be drastically modified, revealing that the crystalline structure is significantly impacted. At 250 °C and above, the peaks disappear, meaning there is no more structuration of the atoms inside the materials. The decomposition of the CNC-Chl is accordingly evidenced and irreversible since the cellulose pattern cannot be seen anymore when the temperature is set back to 25 °C. These results coincide with the TGA.

Transmittance measurements were performed using a handmade heater with a central circular hole of 5 mm diameter is used to heat the samples. We put the sample between two glass plates to ensure the heating temperature uniformity. Thermo couples (type T) were distributed at the interface of the sample and glass plate for measuring the temperature of the sample. A collimated light source of pcW-LEDs is used to shine the sample. The integrating sphere measures transmitted flux. The temperature of the sample is measured by thermal couples at both sides of the sample of silicone plates containing CNC 1.0% of w.t is heated up to different temperatures of 100 °C, 147 °C, 159 °C, 195 °C, and 244 °C, respectively. The thickness of samples is 0.6 mm. The heating duration time is 5 h. The transmission spectrum of the sample at each temperature is calculated as the ratio of transmitted flux at the thermal equivalent temperature to that at the initial temperature. The result of the spectra versus different heated temperatures is shown in Fig. 2c. The results reveal a slight homogeneous absorption at 100 °C, assigned to the CNC-Chl particles. At 159 °C, absorption increases by 8% at a low wavelength and by 4% in the red region. At 195 °C, a 10% absorption is noted in the blue region, with no modification of the red region. Below 200 °C, the average increase of absorption (5–10%) can be linked to a slight modification of the cellulose structure due to the loss of adsorbed water evidenced in the thermograms of Fig. 2a. The more intense absorption in the blue region should be related to the dilatation of the particles noted after XRD analysis. At 244 °C, the absorption increases drastically, especially in the blue region, in line with the CNC-Chl decomposition observed by TGA and XRD analysis. The darkening is almost total in the blue region, while the absorption increase is only 35% in the red region, likely because of the diffusion of small-decomposed fragments^42^. So, it can be concluded that the decomposition of CNC-Chl above 200 °C provoked a non-uniform absorption along the UV–Visible range, which might be related to the nanosize of the chars formed during decomposition. A similar behavior was noted with carbon nanoparticles^43^, that displayed a maximum of absorption at 420 nm, or with carbon dots, for which the absorption increased from 500 to 200 nm^44,45^.

The thermal properties determined by TGA, XRD, and transmittance measurements clearly show that the cellulose nanocrystals functionalized by vinyl chloroacetate can be used as an efficient optical fuse when overheated after dispersion in the silicone matrix.

## CNC’s properties at different operation conditions

According to that application purpose, two requirements should be satisfied to ensure the CNC is suitable material for preventing blue light leakage. The first one is the minor and reversible effect of the CNC on the optical properties at a temperature of less than 150 °C. The second requirement is the irreversibility of darkening at higher temperatures.

In normal working condition of pcW-LEDs, the temperature of the epoxy lens is less than 150 °C, since pcW-LEDs is recommended to operate so that the junction temperature is not over 150 °C. As a result, shown in Fig. 2c, there is a slight decrease in transmission at temperatures less than 150 °C. If it has induced an irreversible darkening of the epoxy lens, this type of CNC material will not be suitable.

**Figure 3:**
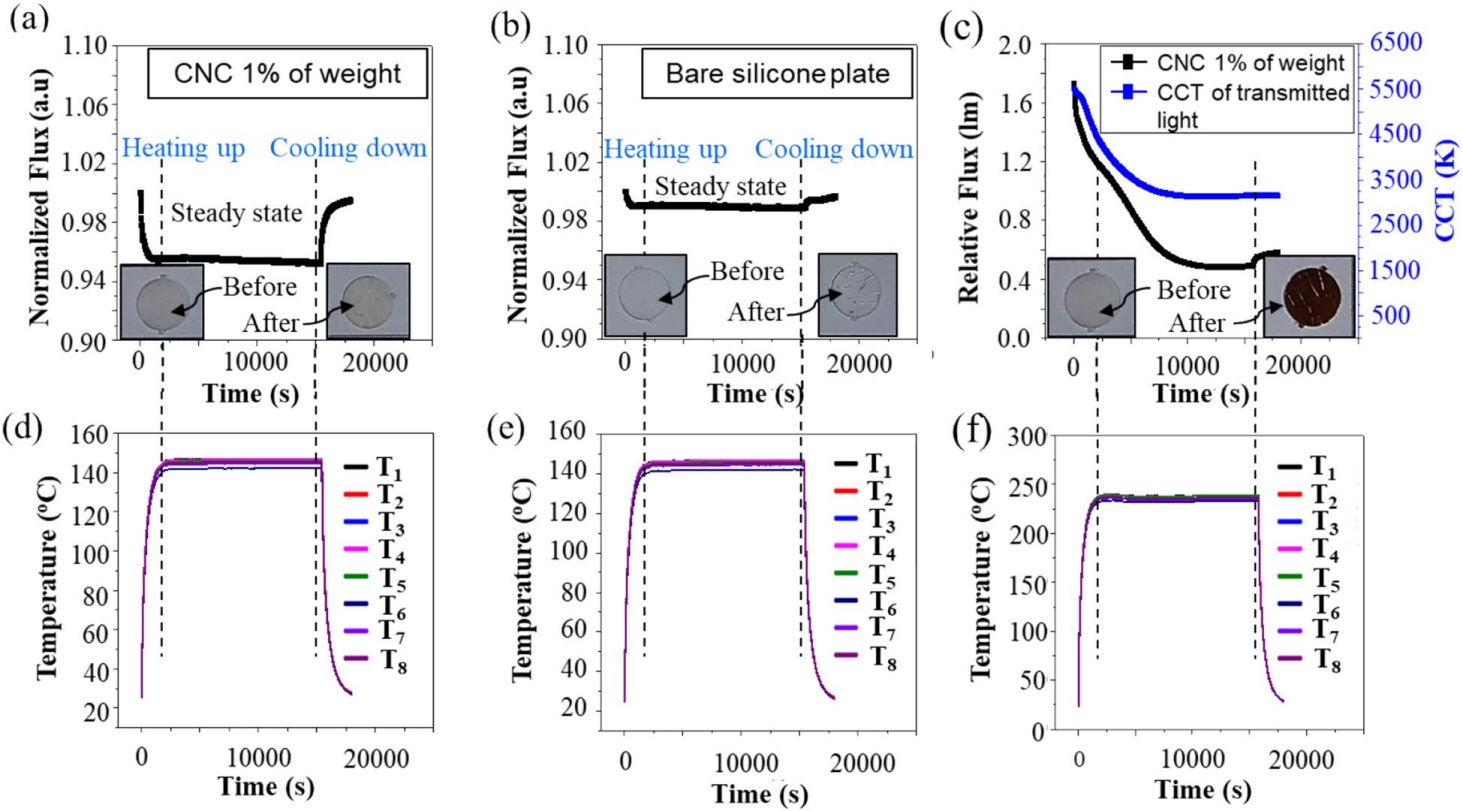
Transmitted flux behavior at stages of heat up, thermal equivalent and cooling down. (**a,d**) The case of the sample of silicone plate containing CNC corresponding to the normal working conditions. (**b,e**) The case of the sample of bare silicone plate corresponding to the normal working conditions. (**c,f**) The case of sample of silicone plate containing CNC corresponding to the abnormal working conditions (T_1_ to T_8_ denotes the thermocouples number one to number eight).

Since testing the reversible property was important, the sample of silicone plate containing CNC 1% of wt. was heated to a temperature of 147 °C, kept at this temperature for five hours, then cooled to room temperature. The behavior of transmitted flux, which corresponds to three states of heating, thermal equivalent, and cooling down, was measured as shown in Fig. 3a while the temperature corresponding was shown in Fig. 3d. The result showed that when the temperature increased from room temperature to around 147 °C, the transmitted flux decreased by 4%. At the thermal equivalence state, the transmitted flux is kept constant. When the temperature decreases to a lower temperature, the transmitted flux is recovered like the initial value. The reversible property of CNC indicated that at a temperature less than 150 °C, the CNC is still not darkened.

For a deeper understanding of this reversible property of CNC, a similar experiment is conducted to understand the effect of temperature on the Silicone-epoxy material. The result is shown in Fig. 3b, e. The results reveal that the same reversible behavior occurs for the bare Silicone sample. However, the decreasing transmitted flux is smaller than 1% than the case sample of silicone plate containing CNC.

An important property of CNC for anti-blue light leakage research is that the CNC should be irreversible after being darkened. It is thus necessary to experiment with checking the irreversible property of CNC. The sample of silicone plate containing CNC 1% of wt. was heated up to 244 °C. When the state of thermal equivalent was established, this heating condition was kept for five hours and then cooled down to room temperature by turning off the power for the heater. The obtained results are shown in Fig. 3c, f. Transmitted flux changing corresponding to three states of heat up, thermal equivalent, and cooling down was shown in Fig. 3c. The corresponding temperature data is shown in Fig. 3f. The big difference in transmitted flux between after cooled and initial transmitted flux indicates that the transparent state of CNC could not be reversible as same as the initial state once darkened. Photo of samples before and after experiments are included in Fig. 3 and show the darkening of the plates heated at 244 °C, and no visible effect for the bare sample and the sample heated at 147 °C.

## Application in real sample pcW-LEDs

**Figure 4:**
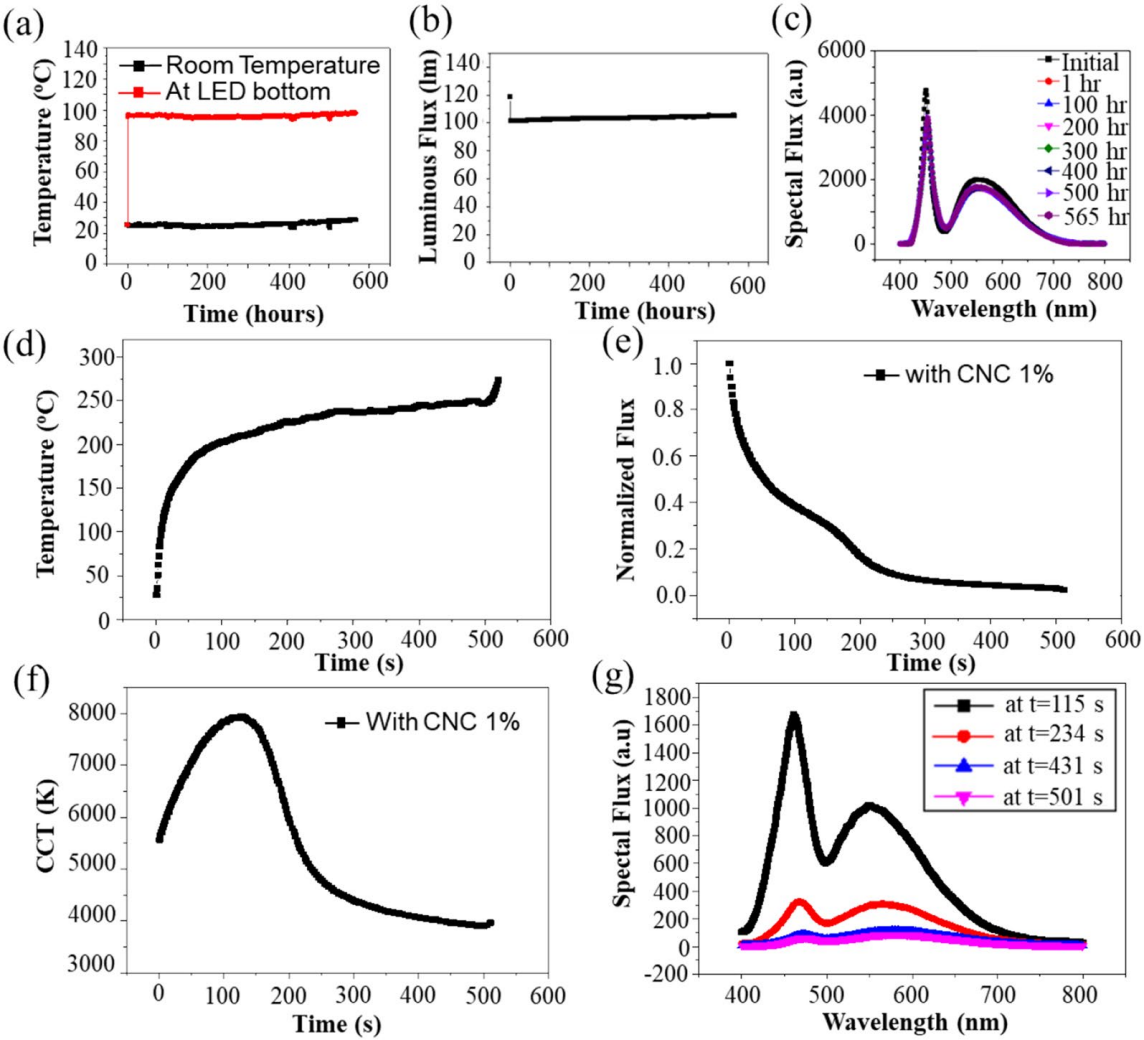
Behavior of (**a**) the temperature measured at the back side of sample and room temperature, (**b**) output luminous flux, and (**c**) output spectra at the normal working condition for long time measurement of 565 h of the sample with CNC 1%. Changing of (**d**) temperature, (**e**) luminous flux, and (**f**) correlated color temperature versus the time when happening the overheat in the sample. (**g**) Changing of spectrums at several selected times corresponding to before and after the darkening of CNC.

The next step is to study in properties of real pcW-LEDs containing CNC in its epoxy lens, as described in Fig. 1. For this purpose, a sample of pcW-LEDs with adding CNC 1% inside to the packaging volume is operating at 0.35A, so the temperature at the back side is about 100 °C. The long-term testing time in the integrating sphere is set at 565 h. The temperature is detected by the thermocouple put at the backside of pcW-LEDs which is put on the glass plate. Thermocouple is connected to the PicoLog TC08 connector, which is controlled by the software in the computer. Each hour, measurements are performed. The tested sample is put inside the integrating sphere. The corresponding experimental result is shown in Fig. 4a–c. Figure 4a is room temperature and temperature at the backside of the sample. The temperature of the backside of the sample is increased from room temperature to 97 °C.

Figure 4b is the luminous flux behavior of output light during the testing time. Luminous is reduced by 15% when the temperature increases from room to thermal-equivalent temperature, then kept constant. The flux behavior indicates that at normal conditions, there is no critical effect on the long-term output luminous flux. Spectra of output flux at several times are measured during the experiment and shown in Fig. 4c. The result shows the highest output flux at the initial time (room temperature). Spectra at times of 1, 100, 200, 300, 400, 500, and 565 h, respectively, are almost overlapped. The CCT calculated on each spectrum are identical, and the corresponding CCT values are 6494 K, 6610 K, 6653 K, 6679 K, 6620 K, 6816 K, and 6580 K, respectively. These spectra indicated that after 565 h of testing at normal working temperature conditions, there was almost no change in the output spectra, which means the CNC has no color degradation at this temperature condition. The results evidence the suitability of CNC in the epoxy lens of pcW-LEDs working at standard conditions.

The final work is to test the efficiency of CNC in blue light leakage prevention. To cause the darkening for CNC in the sample pcW-LEDs with adding CNC, an overheating condition has been generated by the driven sample at high electrical current and poor heat dissipation. The results are shown in Fig. 4d–g. From the initial moment to the 150 s, the overheating caused the thermal degradation in output luminous flux and an increase of CCT. The thermal degradation of luminous flux is caused by increasing nonradiative recombination, which results in generated heat rather than photon generation. After 150 s, when the overheating becomes more serious, the temperature is over 200 °C, and the output luminous flux decays more strongly. (Fig. 4e). a bench in the curve is revealed. This is due to the darkening of CNC). As seen on the luminous spectra (Fig. 4g) and the CCT (Fig. 4f), the decrease in the yellow emission is more significant than the decrease in the blue emission. It reveals that the phosphors are drastically modified during the overheated experiment.

## CNC concentration optimization

**Figure 5:**
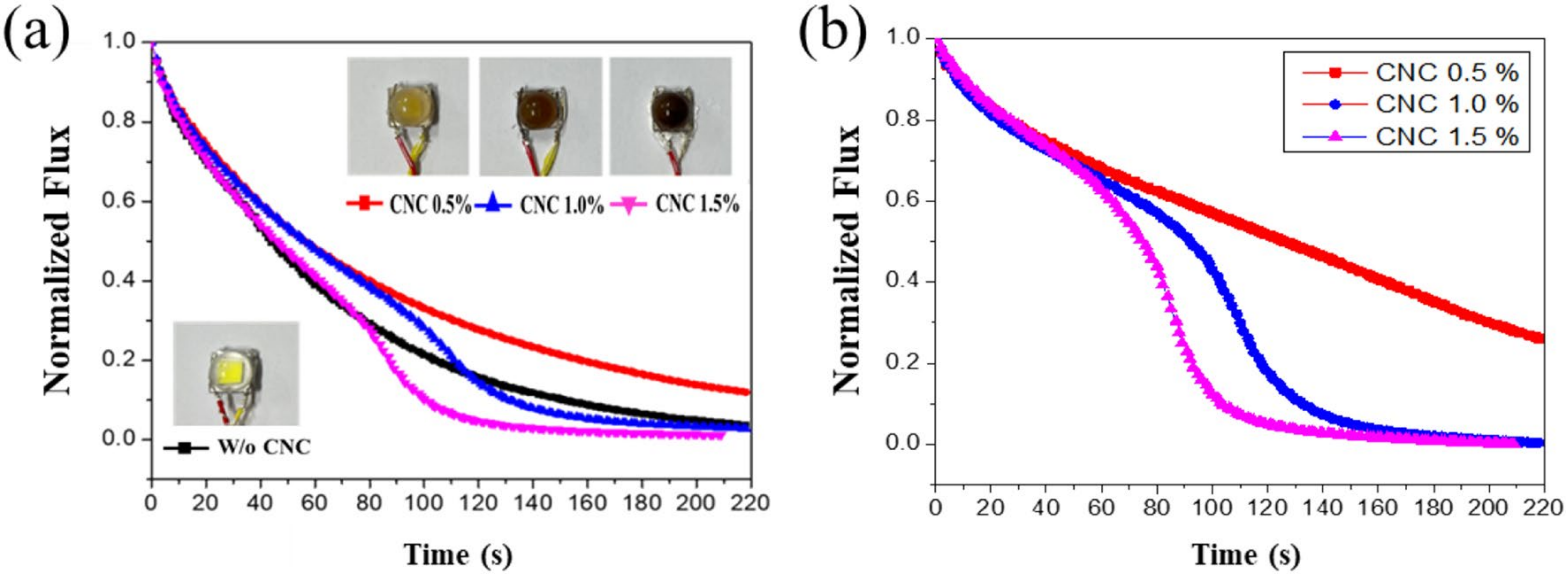
(**a**) The black-color level versus the doped weight concentration of CNC. (**b**) The changing of blue light flux versus time as a function of doped weight concentration of CNC.

The previous sections showed that CNC material is suitable for anti-blue light leakage. However, it still needs to optimize the effect of CNC doping amount on the performance of anti-blue light leakage. Overheating aging tests by the overdriven current of 1.4 A are conducted for pcW-LEDs samples without 0.5%, 1.0%, and 1.5% of CNC in the epoxy lens. Color changing and optical properties will be measured and handled to compare the quenching of luminous flux versus the CNC weight concentration. The visual behavior of the pcW-LEDs sample as a function of the CNC weight concentration is shown in Fig. 5. The darkening level depends on the added CNC weight concentration; the color of samples without CNC shows a clear color of the epoxy lens, while the color samples CNC 0.5%, CNC 1.0%, and CNC 1.5% after aging testing are yellowish, brown, and bold black.

The relative luminous flux is shown in Fig. 5a, where for samples free of CNC and containing 0.5%, the luminous flux regularly decreases, with no bending in the curve. In addition, the luminous flux of the 0.5% sample is higher than the bare silicon sample. The difference in behavior between the sample without CNC (bare Silicone lens) and sample CNC 0.5% may be related to some mechanism, such as the presence of CNC helping increase the light extraction efficiency and improve the thermal management efficiency for the packaging volume^35^.

For samples CNC 1.0%, the bending of the luminous flux curve at the time of 100 s is obvious. The luminous flux curve of sample CNC 1.5% is stronger than that of sample CNC 1.0%, and the bending starts at 80 s. The values of both luminous flux curves of samples CNC 1.0% and CNC 1.5% are less than 0.1 after the bending process, indicating the pcW-LEDs become dimmer. These results indicate that 0.5% of darkened CNC is not enough to absorb the light to cause an essential diming of the pcW-LEDs. However, the concentration of CNC 1.0% and CNC 1.5% are suitable for causing a significant quenching when overheating happens. However, to save the material and be cost-effective, the concentration of 1.0% of CNC is good enough to cause a significant flux quenching.

The changing of blue light flux versus time is shown in Fig. 5b. The bending in the curve of blue light flux is clear and strong for sample CNC 1.0% and CNC 1.5% rather than that of sample CNC 0.5%. The results indicated the amount of absorbed blue light flux depends on of doped weight concentration of CNC. Also, the concentration of 1.0% of CNC is good enough to cause a significant absorption for blue light flux.

## Conclusions

To our best knowledge, a thermal chromic-based unique solution applied for the phenomena of bluish output light for the pcW-LEDs when overheating happen has been proposed and demonstrated for the first time. A pcW-LEDs, when overheating conditions happen, should stop working to help human eyes no longer to exposed to bluish light. To solve that issue, the CNC-Chl has been introduced to the epoxy lens to act as a preventing layer with two properties of transparency at the normal working condition and become irreversibly darkening the abnormal working condition with overheat happens. The property of CNC-Chl material was investigated before being introduced into the packaging volume of pcW-LEDs. The darkening of CNC in the aging testing experiment showed high efficiency in suppressing output flux and CCT. Therefore, the user's eyes are no longer exposed to the bluish light once it appears. The solution is meant to increase lighting quality and safety for human eyes regarding photobiology. Also, an advantage of the darkening of CNC in causing the dimmer of the lamp is to remind the user about the bluish light appearance, so a new one should replace the aging lamp.

## Data Availability

All datasets from this study are available from the corresponding author upon reasonable request.
